# Defects in mitochondrial metabolism and cancer

**DOI:** 10.1186/2049-3002-2-10

**Published:** 2014-07-17

**Authors:** Edoardo Gaude, Christian Frezza

**Affiliations:** 1 Medical Research Council Cancer Unit, University of Cambridge, Hutchison/MRC Research Centre, Cambridge Biomedical Campus, Box 197, Cambridge CB2 0XZ, UK

**Keywords:** Cancer, Metabolism, Mitochondria, TCA cycle, mtDNA mutations, Oncometabolites, Evolution

## Abstract

Cancer is a heterogeneous set of diseases characterized by different molecular and cellular features. Over the past decades, researchers have attempted to grasp the complexity of cancer by mapping the genetic aberrations associated with it. In these efforts, the contribution of mitochondria to the pathogenesis of cancer has tended to be neglected. However, more recently, a growing body of evidence suggests that mitochondria play a key role in cancer. In fact, dysfunctional mitochondria not only contribute to the metabolic reprogramming of cancer cells but they also modulate a plethora of cellular processes involved in tumorigenesis. In this review, we describe the link between mutations to mitochondrial enzymes and tumor formation. We also discuss the hypothesis that mutations to mitochondrial and nuclear DNA could cooperate to promote the survival of cancer cells in an evolving metabolic landscape.

## Review

### Background

Current evidence suggests that the eukaryotic cell originates from the symbiosis between a hydrogen-dependent archaebacterium, the host cell, and a hydrogen-producing eubacterium, the ancestor of modern mitochondria, started two billion years ago [1,2]. This cooperation granted to the newly formed eukaryotic cell several evolutionary advantages, including a more efficient metabolism [1], the detoxification from the harms of the raising levels of atmospheric oxygen [1], and the ability to form multicellular organisms [3]. During evolution, the interaction between mitochondria and the host cell evolved into a more intimate relationship and mitochondria lost control of many of their functions by transferring part of their genome to the nucleus [4]. However, although subordinate to the nucleus, mitochondria maintained the capacity to communicate to the rest of the cells. Mitochondria are in fact the gatekeepers of the eukaryote's cell viability by regulating programmed cell death [5], and they control nuclear functions by the production of reactive oxygen species (ROS), by the modulation of calcium levels [6], and by the trafficking of small molecule metabolites [7]. It is therefore not surprising that the aberrant communication between mitochondria and the rest of the cell may lead to alterations of cellular homeostasis and, in multicellular organisms, to organismal dysfunction. Indeed, altered mitochondrial function has been related to diverse pathological conditions, including cardiovascular disorders, muscular degeneration, neurodegenerative disorders [8], and cancer [9]. Although the connection between mitochondria dysfunction and cancer has historically focused on metabolism [10], their contribution to cell homeostasis goes far beyond metabolism. In this review, we will describe how mitochondrial dysfunction caused by either nuclear or mitochondrial DNA mutations of key metabolic enzymes can initiate a complex cellular reprogramming that supports tumor formation and growth.

### Defects in TCA cycle enzymes and cancer

Among the metabolic pathways that operate in the mitochondria, the tricarboxylic acid (TCA) cycle has recently been in the spotlight of the field of oncology. TCA cycle enzymes are encoded by nuclear DNA (nDNA) and are located in the mitochondrial matrix, with the exception of succinate dehydrogenase, which is embedded in the inner mitochondrial membrane, facing the matrix. In the last decade, several enzymes of the TCA cycle, which we will briefly describe in the following paragraphs, have been found mutated in both sporadic and hereditary forms of cancer.

#### Citrate synthase

**Figure 1:**
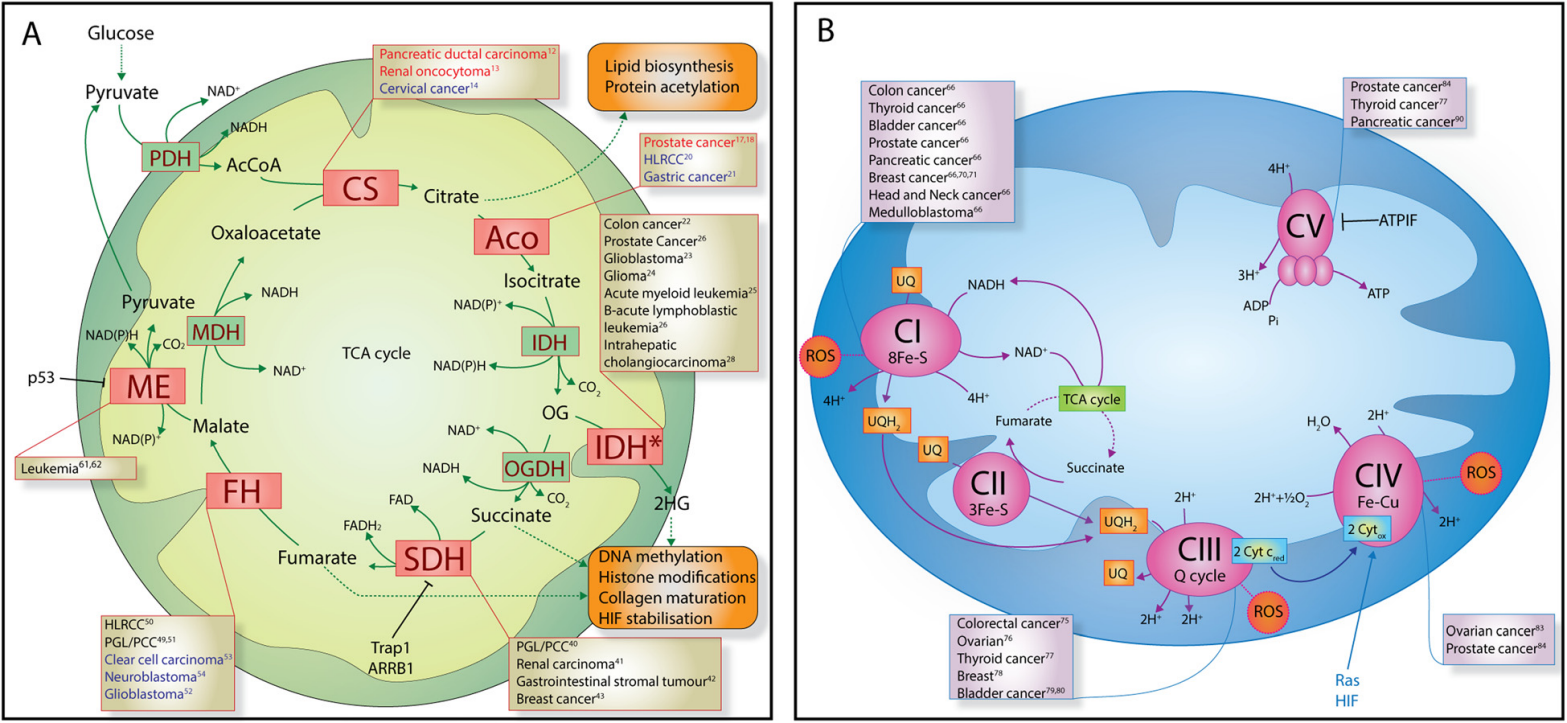
**Mitochondrial dysfunctions in cancer.** Schematic representation of mitochondrial enzymes involved in cancer, focusing on enzymes of the TCA cycle **(A)** and of the respiratory chain and ATP synthase **(B)**. The type of cancer associated with each individual enzyme is listed in boxes. The color of the text indicates if the enzyme has been found upregulated (red), downregulated (blue), or mutated (black) in the given tumor type. *CS* citrate synthase, *Aco* aconitase, *IDH* isocitrate dehydrogenase, IDH* mutant IDH, *OGDH* oxoglutarate dehydrogenase, *SDH* succinate dehydrogenase, *FH* fumarate hydratase, *ME* malic enzyme, *MDH* malate dehydrogenase, *PDH* pyruvate dehydrogenase, *OG* 2-oxoglutarate, *2HG* 2-hydroxyglutarate, *HLRCC* hereditary leiomyomatosis and renal cell cancer, *PGL/PCC* hereditary paraganglioma and pheochromocytoma, *CI–CV* complex I–V, *Cyt c* cytochrome c, *UQ* ubiquinone, *UQH*_*2*_ ubiquinol, *ROS* reactive oxygen species, *ATPIF* ATP synthase inhibitory factor. Dashed lines indicate a series of reaction in a complex pathway, whereas solid lines indicate a single step reaction.

Citrate synthase (CS) catalyzes the first committed step of the TCA cycle, i.e. the irreversible condensation of acetyl coenzyme A (AcCoA) and oxaloacetate into citrate. Citrate can then proceed into the TCA cycle or can be exported to the cytosol and used for protein acetylation or fatty acid biosynthesis [11] (Figure 1A). Evidence for a role of citrate synthase (CS) in cancer is sparse and controversial: CS was found to be increased in pancreatic ductal carcinoma [12] and renal oncocytoma [13] but downregulated in various cervical cancer cell lines [14]. Unfortunately, whether these changes are a simple reflection of variations in mitochondrial mass has not been determined. Furthermore, it is not clear how the deregulation of CS contributes to tumorigenesis. Two scenarios can be hypothesized. On the one hand, increased CS activity, by providing more citrate, could be an advantage for cancer cells that depend on increased fatty acid biosynthesis, such as pancreatic cancer [15]. On the other hand, the loss of CS, by inducing mitochondrial dysfunction could trigger a tumor-supporting glycolytic switch, commonly found in cancer cells. Interestingly, the loss of CS was linked to the induction of the epithelial-to-mesenchymal transition (EMT), suggesting that CS deficiency not only promotes a metabolic rewiring but also indirectly supports cancer cell invasion and metastasis [14].

### Aconitase

Aconitate hydratase or aconitase (Aco) is a Fe-S cluster enzyme that performs the reversible isomerization of citrate to isocitrate via the intermediate *cis*-aconitate (Figure 1A). The role of aconitase in tumor formation has been mainly investigated in the prostate where this enzyme plays an important physiological role. In normal prostate epithelium aconitase activity is inhibited by high levels of zinc, which leads to an extraordinary accumulation of citrate [16]. In prostate cancer, however, aconitase activity is restored, re-establishing citrate oxidation [17] and decreasing fatty acid synthesis [18]. The subsequent decrease in citrate is a key metabolic feature of the transformed epithelium, making citrate a useful *in vivo* marker for discriminating prostate cancer from surrounding healthy regions [19]. In contrast to the tumor-promoting role of aconitase in prostate cancer, the inhibition of this enzyme has been observed in fumarate hydratase (FH)-deficient cancer cell lines. In these cells, the accumulation of the TCA cycle intermediate fumarate causes the inactivation of the iron-sulfur cluster of the enzyme, leading to a complete loss of aconitase activity (see paragraph on fumarate hydratase (FH) and [20]). Decreased expression of aconitase has also been observed in gastric cancer, and its expression is a prognostic marker of disease progression [21]. Whether mitochondrial aconitase has additional roles beyond regulating citrate availability is currently unknown.

### Isocitrate dehydrogenase

Isocitrate dehydrogenase (IDH) catalyzes the reversible conversion of isocitrate into 2-oxoglutarate (OG). In eukaryotes, one nicotinamide adenine dinucleotide (NADH)-dependent (IDH3) and two nicotinamide adenine dinucleotide phosphate (NADPH)-dependent (IDH1 and IDH2) isoforms of IDH exist (Figure 1A). Mutations of both the cytoplasmic (IDH1) and the mitochondrial (IDH2) NADPH-dependent isoforms have been found in various human cancers, including colon cancer [22], glioblastoma [23], glioma [24], acute myeloid leukemia [25], prostate cancer [26], B-acute lymphoblastic leukemia [26], osteosarcoma [27], and intrahepatic cholangiocarcinoma [28]. Oncogenic mutations confer a neomorphic activity to IDHs, which instead of converting isocitrate in OG, reduce OG into the R-enantiomer of 2-hydroxyglutarate (R-2HG), which accumulates up to millimolar levels in cancer cells (See Figure 1A and [29,30]). This poorly characterized metabolite is now considered a major contributor to the oncogenic activity of mutated IDHs. Indeed, the incubation of cells with R-2HG promotes cytokine independency and blocks differentiation in hematopoietic cells, inducing leukemogenesis [31]. The tumorigenic activity of 2HG has been attributed to its inhibitory effect on various OG-dependent dioxygenases, including the hypoxia-inducible factors (HIFs) prolyl hydroxylases (PHDs), histone demethylases, and the ten-eleven translocation (TET) family of DNA demethylases [32,33]. The first evidence that 2HG acted upon DNA methylation arose in 2010 when a large-scale DNA methylation analysis of human leukemia found that the expression of mutated IDH, by increasing 2-HG levels, led to DNA hyper-methylation, a broad epigenetic change associated with poor hematopoietic differentiation. Of note, such a peculiar change in DNA methylation was dependent on the inhibition of TET2 caused by 2HG [34]. A similar epigenetic fingerprint has also been observed in a subset of breast tumors where 2HG was found to accumulate to millimolar levels. Interestingly, however, in these tumors, the accumulation of 2HG was not caused by overt IDH mutations but, rather, by a particular metabolic rewiring instigated by Myc overexpression [35]. These results suggest that 2HG has an important role in tumorigenesis and that it can accumulate in cancer cells not only upon IDH mutations but also as a consequence of metabolic derangements, including hypoxia [36]. More recent results revealed that, besides inhibiting DNA demethylases, 2HG accumulation also causes profound changes in histone methylation [37], indicating that this metabolite has multiple and well-defined epigenetic roles. The inhibitory effects of 2HG toward PHDs are instead more controversial and appear isomer-specific. In fact, while the S-enantiomer of 2HG (S-2HG) was shown to inhibit PHDs, R-2HG activates them, leading to accelerated degradation of HIFs [38]. Although initially unclear, the paradoxical activation of PHDs by R-2HG can be explained by its non-enzymatic oxidation to OG, the natural substrate of these enzymes [39]. Of note, these results imply that HIF is not required for R-2HG-induced tumorigenesis and, on the other hand, suggest that this transcription factor might act as a tumor suppressor in this specific context.

#### Succinate dehydrogenase

Succinate dehydrogenase (SDH) is an enzyme complex bound to the inner mitochondrial membrane that converts succinate into fumarate, in a reaction coupled to the reduction of flavin adenine dinucleotide (FAD) to FADH_2_. SDH represents a unique link between the TCA cycle and the mitochondrial respiratory chain, where it is also known as respiratory chain complex II (Figure 1A,B). SDH is the only known enzyme of the respiratory chain completely encoded by nDNA and is devoid of proton pumping activity. Inactivating mutations of SDH subunits and assembly factors have been linked to different types of hereditary and sporadic forms of cancer, including hereditary paraganglioma and pheochromocytoma (PGC/PCC) [40], renal carcinoma [41], gastrointestinal stromal tumor [42], and breast cancer [43]. SDH can behave as a classic tumor suppressor gene since the mutated allele is inherited in a heterozygous fashion, while the remaining wild type allele is lost in tumor samples. Similarly to mutant IDHs, most of the oncogenic activity of SDH mutations has been attributed to a metabolite, succinate, which accumulates in SDH-deficient cells. The oncogenic role of succinate was initially linked to the inhibition of PHDs and the subsequent stabilization of HIF [44]. More recently, succinate was found to be a prototypical ‘epigenetic hacker’ [45], capable of inhibiting both DNA [46,47] and histone demethylases [48], leading to epigenetic changes that overlap with those observed in mutant IDH cancers [49].

### Fumarate hydratase

FH catalyzes the reversible conversion of fumarate to malate (Figure 1A). Germline mutations of FH were originally discovered in hereditary leiomyomatosis and renal cell cancer (HLRCC) [50]. More recently FH germline mutations were also found in a subset of PGC/PCC [49,51]. FH was also found to be downregulated in glioblastoma [52] and sporadic clear cell carcinoma [53] and deleted in non-Myc-amplified neuroblastoma [54]. Similarly to SDH, FH behaves as a classic tumor suppressor. Part of its tumorigenic activity has been attributed to the abnormal accumulation of fumarate, which peaks to high millimolar levels in FH-deficient cancer cells [55]. Fumarate shares some similarities with succinate and 2HG in that it can inhibit several OG-dependent enzymes, including PHDs [56], and histone and DNA demethylases [46]. Interestingly, however, fumarate possesses another unique property linked to its chemical structure. In fact, fumarate is a moderately reactive α,β-unsaturated electrophilic metabolite that, under physiological conditions, can covalently bind to cysteine residues of proteins in a process called *succination*[57,58]. Several proteins are succinated in FH-deficient cells, including aconitase [20], and Kelch-like ECH-associated protein 1 (Keap1) [57,58]. Of note, the succination of Keap1 abrogates its inhibitory activity toward the nuclear factor (erythroid-derived 2)-like 2 (Nrf2) transcription factor, leading to the activation of several antioxidant genes thought to play key roles in supporting tumor formation [57,58]. Interestingly, also, the reactive thiol residue of GSH is subject to succination, and this phenomenon is linked to increased oxidative stress in FH-deficient cancer cells UOK262 [59].

#### Malic enzyme

Malic enzyme (ME) catalyzes the oxidative decarboxylation of malate into pyruvate and CO_2_ (Figure 1A). In mammalian cells, two NADP^+^-dependent MEs, the cytosolic ME1 and the mitochondrial ME3, and the mitochondrial NAD^+^-dependent ME2 have been described. The first link between mitochondrial MEs and cancer traces back to the 1970s, when Lehninger's laboratory observed that mitochondria isolated from leukemia-derived ascites cancer cells carried unexpectedly high rates of conversion of malate into pyruvate [60]. Ten years later, the same lab suggested that malate metabolism is compartmentalized: malate generated from glutamine oxidation in the mitochondria proceeds through the TCA cycle, whereas cytosolic malate is converted into pyruvate by the mitochondrial ME2. The authors also observed that extra-mitochondrial malate, after conversion into pyruvate and then citrate, could fuel fatty acids and cholesterol biosynthesis, supporting tumor growth [61]. More recent evidence underscored the role of this enzyme in leukemia cells, where the silencing of ME2 led to diminished proliferation and increased apoptosis [62]. Interestingly, the expression of ME1 and ME2 has been found to be regulated by p53 and to tightly control NADPH homeostasis, corroborating the connection between these enzymes and oncogenic metabolic rewiring [63].

### Mitochondrial DNA mutations and cancer

Mitochondria contain a circular chromosome of 16,596 base pairs, coding for 37 genes translated into 13 subunits of the respiratory chain and ATPase complexes, 22 tRNAs and 12S and 16S ribosomal RNAs. Mammalian cells contain thousands of copies of mitochondrial DNA (mtDNA) [64]. In contrast to nDNA, mtDNA mutations coexist with normal mtDNA in a heterogeneous mixture known as heteroplasmy. Importantly, by varying the level of heteroplasmy, a single mtDNA mutation might result in a wide range of bioenergetics defects, from mild mitochondrial dysfunction to a severe bioenergetic impairment and cell death [65]. Somatic mtDNA mutations have been found in a wide array of human cancers including tumors of colon, breast, lung, prostate, liver, pancreas, kidney, thyroid and brain as well as in gastric carcinoma and ovarian cancer [66] and are usually associated with bioenergetics defects. Nevertheless, a complete loss of mtDNA seems detrimental for cancer cells. For instance, experiments with mtDNA-deficient cells (*ρ*^0^ cells) have clearly shown that cancer cells need functional mitochondria for their survival and proliferation [67,68]. A thorough description of mtDNA mutations in cancer has been given in other excellent reviews (see for instance [66] and [9]). In our review, we will summarize the most recent findings and propose a unifying theory of the role of mtDNA mutations in cancer.

#### Complex I

Among mtDNA mutations associated with cancer initiation and progression, those affecting complex I (CI) of the respiratory chain are the most common. CI, also known as NADH:ubiquinone oxidoreductase, catalyzes the transfer of two electrons from NADH to ubiquinone via flavin mononucleotides, producing NAD^+^ and four protons, which are pumped in the intermembrane space (Figure 1B) [11]. CI is the first site of the electron transport chain and active site of reactive oxygen species (ROS) production. Therefore, mutations in CI can significantly alter cell bioenergetics and redox homeostasis [69]. Mutations in mitochondrial genes encoding for CI have been linked to the development of colon, thyroid, pancreas, breast, bladder, and prostate cancer as well as of head and neck tumors and medulloblastoma (reviewed in [66]). Furthermore, mtDNA mutations that affect CI have been linked to increased ROS-dependent metastatic potential in Lewis lung carcinoma and breast cancer cells [70,71]. The contribution of CI mutations to cancer largely depends on the corresponding bioenergetics dysfunction that they cause. In fact, cancer cells affected by severe CI deficiency exhibited decreased tumorigenic potential both *in vitro* and *in vivo*, if compared to cells with a mild CI dysfunction [72] and CI activity is required for the induction of aerobic glycolysis in osteosarcoma cells [73]. In line with these finding, a recent study showed that intact CI activity is essential for cancer cell survival at low glucose levels, a condition commonly found in tumor microenvironment [74].

#### Complex III

Complex III, also known as coenzyme Q:cytochrome *c* oxidoreductase, or cytochrome bc1, catalyzes the electron transfer from reduced ubiquinone or coenzyme Q 10 to cytochrome *c* followed by the pumping of four protons into the intermembrane space (Figure 1B). mtDNA mutations that affect CIII have been found in various cancers, including colorectal [75], ovarian [76], thyroid [77], breast [78], and bladder [79] cancers. In support to an oncogenic function of CIII dysfunctions, it was demonstrated that the expression of a truncated subunit of CIII in MB49 bladder cancer cells increases cell growth and invasion both *in vitro* and *in vivo*[80]. Interestingly, this oncogenic phenotype was accompanied by lactate secretion, increased ROS production, and resistance to apoptosis via activation of NF-κB2 pathway [80]. In line with these findings, the expression of a mutated form of CYTB in SV40-immortalized human uroepithelial cells induced an antiapoptotic signaling cascade that sustained cancer cell growth [81]. Together, these results suggest that mtDNA mutations that affect CIII activity are sufficient to drive tumorigenesis via a mechanism that involves ROS production and the inhibition of apoptosis.

#### Complex IV

Cytochrome *c* oxidase, also known as complex IV (CIV) is the terminal complex of the respiratory chain. CIV is composed of 12 subunits, of which 3 (I, II, and III) are encoded by mtDNA and 9 (IV–XIII) by nDNA. CIV receives four electrons from cytochrome *c* and reduces molecular oxygen into water and four protons, which are pumped in the intermembrane space (Figure 1B). CIV is the rate-limiting step of respiratory chain and a well-characterized site of ROS production [82]. The link between CIV activity and cancer is controversial. Mutations of the mtDNA-encoded CIV subunit 1 (COX1) have been associated with ovarian cancer [83] and prostate cancer [84]. On the other hand, nDNA-encoded subunits of CIV are generally upregulated in cancer. For instance, the overexpression of the antiapoptotic protein Bcl-2 in leukemia cells increased the mitochondrial localization of the subunit Va of CIV (cytochrome oxidase (COX) Va) and COX Vb, leading to increased respiration and high intracellular ROS [85]. In line with these findings, the expression of oncogenic Ras in immortalized human bronchial epithelial cells increases CIV activity and the inhibition of Ras in A549 lung adenocarcinoma cells reduces COX Vb expression [86]. Finally, hypoxia, an environmental cue experienced by cancer cells, can also increase CIV efficiency by regulating the ratio between two CIV subunits (COX4-1 and COX4-2) in HIF1-dependent fashion [87]. These results seem to suggest that mtDNA-encoded subunits are generally tumor-suppressing, whereas nDNA encoded-subunits are tumor-promoting.

#### Complex V

Adenosine triphosphate (ATP) synthase, also known as complex V (CV), is the final enzyme of oxidative phosphorylation. CV exploits the electrochemical potential gradient across the inner mitochondrial membrane to generate ATP from ADP and inorganic phosphate (Figure 1B). Of note, the ATP synthase has recently been found to be part of the permeability transition pore (PTP) [88], a membrane-embedded mitochondrial complex involved in several mitochondria-dependent processes, including calcium buffering and apoptosis [89]. Mutations in CV subunits encoded by mtDNA have been found in thyroid [77], pancreatic [90], and prostate [84] cancer. To investigate the oncogenic activity of CV mutations, Shidara and colleagues introduced two different point mutations in the mtDNA gene encoding for the CV subunit 6 (MTATP6) [91]. Interestingly, mutant ATP6 increased cell proliferation in 2D cultures and led to higher oncogenic potential in xenografts. Importantly, the reintroduction of a nuclear-encoded wild-type ATP6 suppressed tumor formation in these cells. Several factors could explain the link between CV mutations and tumorigenesis. For instance, mutant cells displayed reduced apoptosis, suggesting that the oncogenic function of mutant ATP6 could involve inhibition of programmed cell death, which is consistent with the role of CV in the regulation of the PTP [88]. Also, ATP6 mutations were associated with increased ROS production, suggesting that, even if the ATP synthase is not directly involved in the transport of electrons, its inhibition could cause electron leak from the respiratory chain, inducing ROS generation. In contrast with the link between low CV and cancer, a recent work showed that a functional ATP synthase is instead required for cell survival in the presence of overt dysfunction of oxidative phosphorylation. Indeed, it was recently found that the loss of the ATPase inhibitory factor ATPIF1 protected from antimycin-induced cell death, in a human haploid cells. Interestingly, it was demonstrated that the ablation of ATPIF1 is required to allow the reversal of ATP synthase, a process whereby ATP synthase hydrolyses ATP to maintain a mitochondrial membrane potential [92]. These observations underscore the plasticity of CV, which can shape its activity to maintain mitochondrial potential and, eventually, to support survival.

## Conclusions

**Figure 2:**
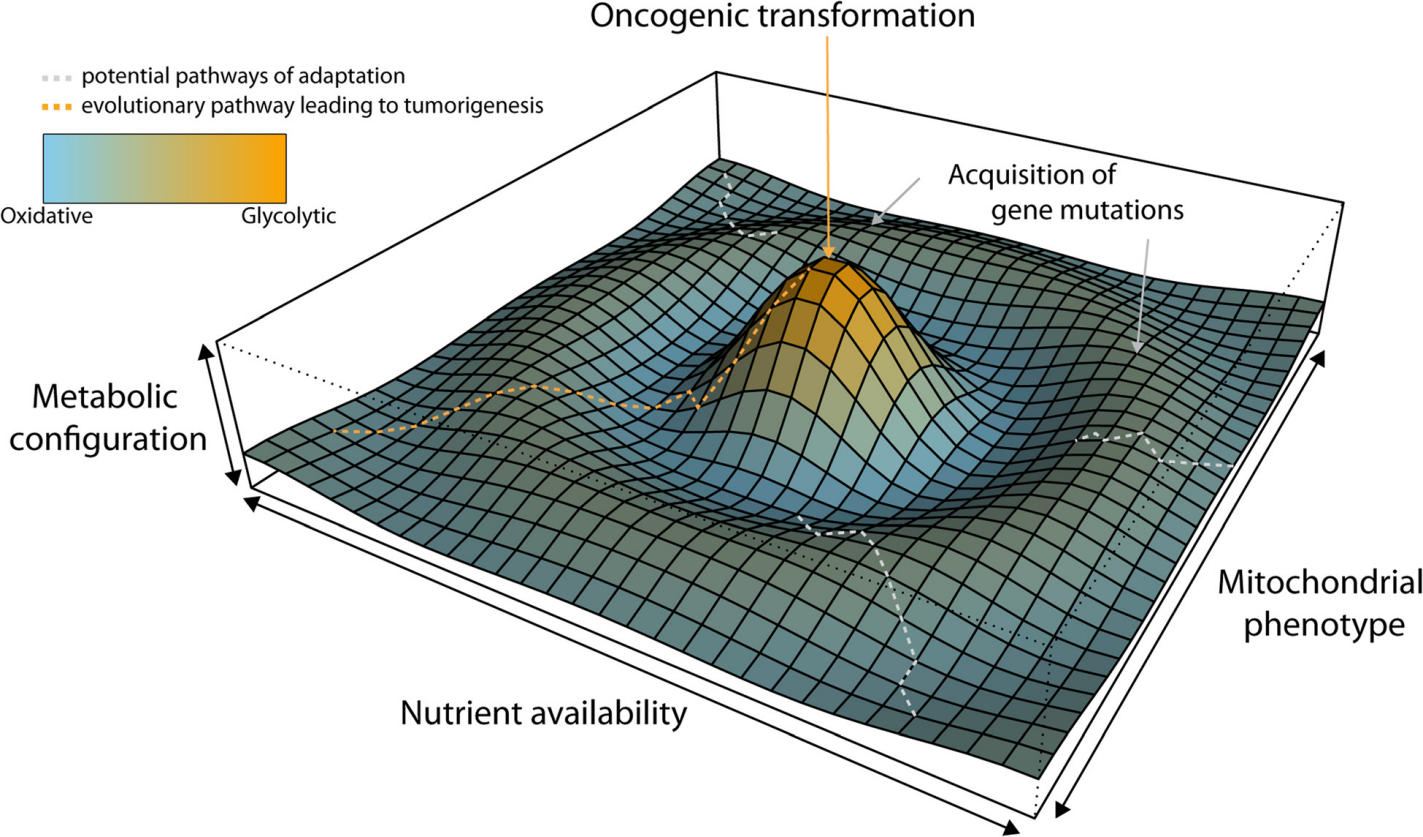
**The evolving metabolic landscape of a cell.** Schematic representation of the evolutionary process of a cancer cell driven by metabolic cues. The high bioenergetic flexibility of mitochondria allows cells to adapt to ever-changing environments, acquiring different metabolic configurations within the metabolic landscape. This metabolic flexibility is achieved by mutations of mtDNA and further shaped by the degree of heteroplasmy of the mutations itself. According to pre-existing metabolic adaptations (mitochondrial phenotypes) and to nutrient availability, there might be a selective pressure on the acquisition of genetic mutations that can sustain a certain metabolic configuration (gray dashed lines). The nDNA mutation is then passed to the progeny. The fixation of a specific metabolic configuration (e.g. aerobic glycolysis) could then lead to tumorigenic transformation (orange dashed lines) by yet unidentified mechanisms. This scenario could be used to trace the metabolic evolution of cancer based on an evolving metabolic landscape.

In this review, we have explored the link between defects in mitochondrial metabolism, caused by mtDNA or nDNA mutations, and tumorigenesis. We have also discussed the hypothesis that mitochondrial dysfunction not only perturbs cellular bioenergetics, supporting the metabolic transformation of cancer cell, but that it also triggers tumor-promoting (epi)genetic changes mediated by the small molecule metabolites that they release. Given the importance of mitochondria in tumorigenesis, it is not surprising that canonical oncogenes and tumor suppressors exert their functions by regulating mitochondrial function [7]. For instance, Trap1 [93] and the endocytic adaptor protein β-arrestin [94] were shown to alter SDH expression and activate a succinate-dependent pseudoxypoxic response in support of their tumorigenic program. Hence, deregulation of mitochondrial function plays a key role not only in tumor initiation but also during tumor progression, where secondary mitochondrial dysfunction would enable cancer cells to adapt to a constantly evolving tumor microenvironment. In this scenario, however, mtDNA mutations, by virtue of their tunable bioenergetic outcome, would represent a more efficient way to adapt to novel metabolic niches than nDNA mutations. We propose that nDNA and mtDNA mutations are co-selected to finely shape the metabolic efficiency of cancer cell during tumor evolution: mtDNA mutations would enable fast and reversible explorations of different metabolic niches, whereas nDNA mutations would permanently fix an advantageous metabolic configuration and pass this information to the daughter cells (Figure 2). Considering the long-standing evolutionary cooperation between mitochondria and the host cells, it is not surprising that their two genomes are hard-wired for cell survival and proliferation.

## Abbreviations

2HG: 2-hydroxyglutarate; AcCoA: acetyl coenzyme A; Aco: aconitase; ADP: adenosine diphosphate; ATP: adenosine triphosphate; ATPIF: ATPase inhibitory factor; CI–V: respiratory chain complex I–V; CS: citrate synthase; COX: cytochrome oxidase; CYT: cytochrome; EMT: epithelial to mesenchymal transition; FAD: flavin adenine dinucleotide; FH: fumarate hydratase; GSH: reduced glutathione; HIF: hypoxia-inducible factor; HLRCC: hereditary leiomyomatosis and renal cell cancer; IDH: isocitrate dehydrogenase; Keap1: Kelch-like ECH-associated protein 1; ME: malic enzyme; mtDNA: mitochondrial DNA; NADH: nicotinamide adenine dinucleotide; NADPH: nicotinamide adenine dinucleotide phosphate; nDNA: nuclear DNA; Nrf2: nuclear factor (erythroid-derived 2)-like 2; OG: 2-oxoglutarate; PGC/PCC: hereditary paraganglioma and pheochromocytoma; PHD: prolyl hydroxylases; PTP: permeability transition pore; ROS: reactive oxygen species; SDH: succinate dehydrogenase; TCA: tricarboxylic acid; TET: ten-eleven translocation.

## Competing interests

The authors declare that they have no competing interests.

## Authors’ contributions

EG wrote the manuscript and prepared the figures. CF supervised the design of the review and wrote the manuscript. Both authors read and approved the final manuscript.

## Authors’ information

EG is a PhD student of the University of Cambridge in the laboratory of CF. CF is a group leader at the MRC Cancer Unit.
